# Development and Effects of Cognitive Behavior-Based Healing Programs Using Mobile Apps

**DOI:** 10.3390/ijerph18073334

**Published:** 2021-03-24

**Authors:** Won Ju Hwang, Hyun Hee Jo

**Affiliations:** College of Nursing Science, Kyung Hee University, 26 Kyunghee-Daero, Dongdaemun-Gu, Seoul 02447, Korea

**Keywords:** stress management, mental health, mobile health application, mental wellness

## Abstract

Purpose: There has been a recent surge in interest in mental health and how to improve individuals’ health-related quality of life. Mental health management using mobile apps can be a useful intervention method. The development and application of verified and highly efficient apps for mental health and stress management are needed. We developed healing programs and verified their effectiveness based on apps designed to promote adult mental health. Methods: We conducted a one-group pretest-posttest study in which 85 participants used the app for 12 weeks. We assessed its effects on participants’ stress (perceived stress scale, effort–reward imbalance, and photoplethysmogram (PPG)), anxiety, depression, emotional labor, and well-being. Results: The stress survey results post-intervention increased or stayed the same; however, the PPG results decreased (*p* = 0.002) after using the app. Depression (*p* = 0.043) and anxiety (*p* = 0.003) also decreased; however, emotional labor and well-being remained unchanged. The awareness of, knowledge of, and attitude toward mental health management all increased post-intervention. Discussion: The developed mobile app was an efficient and highly accessible way to promote mental health. However, the app requires modification and supplementation for continual use. Additionally, ongoing research concerning the study, evaluation, and integration of mobile apps is required.

## 1. Introduction

According to the report of the Ministry of Health & Welfare [1], one out of every four adults in Korea experiences mental health problems more than once in their lives. In particular, 73.3% of adult workers were found to be under severe stress [2]. Stress causes several physical and psychological problems, such as cardiovascular disease, stomach ulcers, decreased immunity, and depression [3]. According to a mental health awareness survey conducted with Seoul citizens, 48.3% of the respondents experienced mental problems such as stress, depression, and suicidal thoughts in the past year; their way of coping with such problems was to talk to family and friends (47.9%) or try to take care of it themselves (27.3%). Most respondents (91.3%) recognized that mental illness needed medical treatment, just like other diseases; but they experienced difficulties in using medical and counseling institutions [1,4].

With the rapid distribution of smartphones in recent years, smartphone users make up a significant portion of the world’s population and do not leave home without their smartphones [5]. According to an 2019 Internet Survey, people use their smartphones for video and artificial intelligence services, as well as for Internet banking, shopping, and instant messaging [6]. As such, there is a convergence of various electronic devices and services that have evolved recently, which has had a significant impact not only on our daily lives but also on education, labor, industry, and social phenomena [7]. Smartphone users check their mobile phones 150 times a day and use them for more than three hours a day [8]. This explains how smartphones can help form habits and have a powerful effect on maintaining them [9,10].

Mental health can also be improved by using mobile apps [4]. Mental health-related mobile apps are referred to as mental health apps (MHapps), and there are various types [5]. However, several apps are not medically based and are for commercial purposes, and it is believed that unverified MHapps will continue to be developed and distributed. To manage the mental health of the Korean population efficiently, the development and application of effective and reliable MHapps is essential. In particular, mental health professionals should lead the research, evaluation, and integration of mobile apps [11].

Several MHapps have been developed recently, making them available to smartphone users. This plays a key role in future mental healthcare and makes it easy to use mental health resources. They increase temporal, economic, and geographic accessibility so that people can recognize their mental health problems and ask for help [5]. Smartphones are very useful and attractive for protecting personal information and preventing social stigma because they are used in isolation and without spatial restrictions. Downloading and using MHapps also meets the needs of young people and multiple users. Smartphone apps are accessible in almost any environment since users can access them any time [12]. In this respect, research on the use of mobile apps that support diagnosis and management of mental health conditions, as well as treatment and counseling services for mental illness, can be a useful intervention measure to promote the mental health of Koreans.

Given the above circumstances, mobile apps to promote mental health are urgently needed. MHapps for reducing stress can be effectively used at workplaces without constraints of time and place. However, efforts to promote the use of MHapps are still insufficient in Korea. Compared to MHapps in the U.S. and the U.K., there are only a few in Korea number, and those that do exist are not used often [13]. In addition, despite high stress, Koreans are less interested in mental health than physical health [14]. As symptoms of stress, anxiety, and depression due to COVID-19 increase, there are the needs for a shift in perception of mental health for which the provision of services increases [15]. Hence, an intervention method using smartphone apps is believed to be helpful by providing highly accessible mental health services to several people.

In sum, we devised a MHapp to help reduce negative emotions such as stress, depression, and anxiety. In this paper, we will describe the development process of the mobile app; then, we will explain the application and results of the app and discuss its effectiveness.

## 2. Materials and Methods

### 2.1. App Development Process

#### 2.1.1. Structural Design

**Figure 1:**
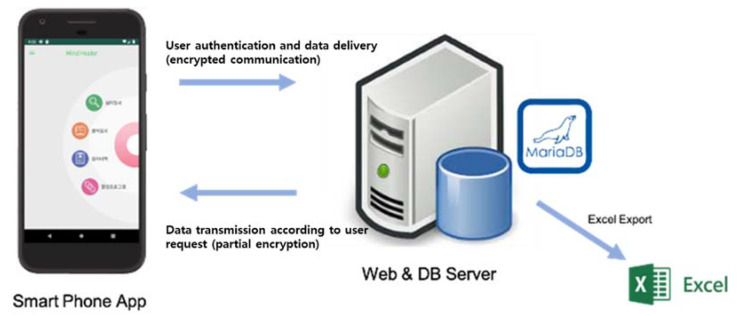
System configuration in database development environment.

It was designed so that, when using an app, the data of log-ins, test results, frequency and type of use, etc., would be accumulated. The database was created by building a Java-based development environment. The tools used were VSCODE (Microsoft Corporation, Redmond, WA, USA), GIT (GIT, New York, NY, USA), MySQL Workbench (Oracle Corporation, Austin, TX, USA), Cyberduck FTP (Cyberduck, Boston, MA, USA), and Safari and Chrome browsers. Development languages were PHP v7.3, HTML, Javascript, and CSS. The database development environment was MariaDB v10 (MariaDB Corporation, Redwood City, CA, USA), and it was developed on a MAC OS. The Linux server (Red Hat, Raleigh, NC, USA) was used, and the goal was to store all data on the server and accumulate big data. The server receives and stores data from smart devices, which is the basis for analyzing data to identify mental health and other conditions and provide customized services (Figure 1).

#### 2.1.2. User Interface Design

**Figure 2:**
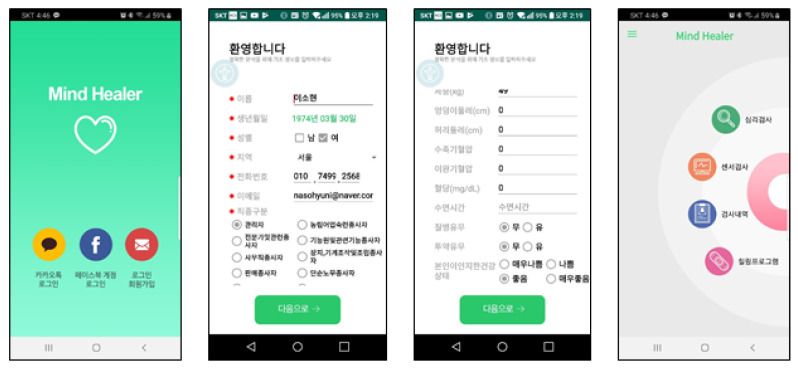
Basic composition of the Mind Healer App. Login screen/Input page of Personal information/Input page of perceived health status/Psychological test and sensor test page.

Figure 2 shows the main screen of Mind Healer, which consists of a psychological test, a sensor test, and a healing program that identify users’ condition. It was designed so that information can be entered to identify users’ health-related information and general characteristics.

**Figure 3:**
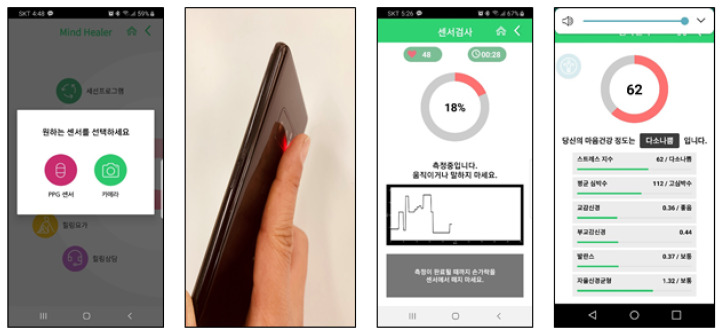
Photoplethysmogram (camera sensor for smartphones) sensor measurement and results. PPG and camera sensor option/Camera sensor apply/measurement/display of PPG.

**Figure 4:**
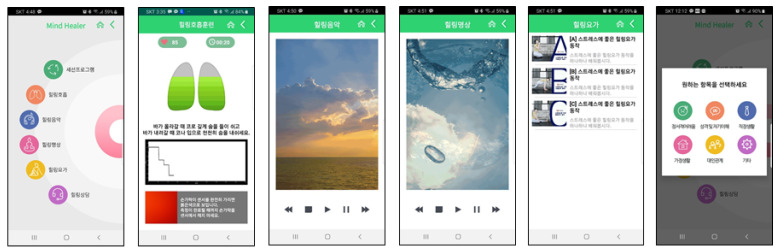
Details of the healing program. Healing program options/breathing/meditation/music/yoga/counseling and recommended program.

(1) There were six types of psychological tests for stress, effort–reward imbalance, depression, anxiety, emotional labor, and well-being. When each test item was clicked and the questions were answered, a score was provided and stored on the app and on the Internet.

- Two tools were used for stress. The first was the Perceived Stress Scale (PSS), developed by Cohen, Kamarck, and Mermelstein [16]. It is a Korean version of the PSS (PS-10)—a tool developed to assess the awareness of subjective stress. It comprises six items that measure negative awareness and four items that measure positive awareness. It evaluates the past month and provides a score from 0 to 4 per item (range = 0–40), and higher scores indicate greater stress awareness. Cronbach’s α was 0.89. Second, we used the effort–reward imbalance (ERI), a tool translated by Hwang, Hong, and Kang [17] based on a tool developed by Siegrist [18]. The ERI measures work-related stress, and Cronbach’s α was 0.80.
- Depression was based on the Patient Health Questionnaire-9 (PHQ-9) score, a self-reporting evaluation measure developed by Kroenke, et al. [19]. We used the Korean version of PHQ-9 by Lee, et al. [20]. Participants were to answer the level of symptoms experienced in the past two weeks based on frequency. Scores ranged from 0 to 27, and higher scores indicated higher depression levels. Cronbach’s α was 0.89.
- Anxiety was measured with the General Anxiety Disorder-7 (GAD-7), a seven-item self-reporting assessment tool developed by Spitzer, et al. [21]. The GAD-7 was designed based on Diagnostic and Statistical Manual of Mental Disorders (DSM)-IV diagnosis criteria to enable a GAD diagnosis. Cronbach’s α was 0.92.
- Emotional labor was measured with the Korean Emotional Labor Scale (K-ELS) by Lee, et al. [22]. It was designed to quantitatively and objectively evaluate the level and intensity of emotional labor and the negative emotional responses caused by emotional labor, which reflects the specific nature of Korea’s organizational culture and service industry. The K-ELS consists of 24 questions measured on a 4-point scale. Cronbach’s α was 0.79.
- Well-being was based on the WHO-5 well-being score. This was measured with five questions on a 6-point scale covering the past two weeks. Higher scores indicate better well-being [23].

(2) A biometric index sensor test was conducted to determine the stress measurement and mental and physical relaxation functions using photoplethysmogram (PPG) sensor or camera sensor which installed in the smartphone. The PPG sensor is a device that projects light with a particular wavelength onto the measured object, detects the light that is reflected off or goes through it, and detects the pulse signal from the user. Then, it converts the detected pulse signals into digital data to define stress indices by using pulse measurement methods (Figure 3) [24]. As a technical method of measuring and analyzing heart rate, the stress index is measured by grasping the average heart rate, sympathetic, parasympathetic, and autonomous balance [25,26]. PPG is a simple and inexpensive optical technology that is efficient for calculating pulse rate variability (PRV) [27,28]. Measurement of heart rate variability (HRV) is known to have information on very-low-frequency (VLF) and low-frequency (LF) sympathetic nerves, and high-frequency (HF) parasympathetic nervous system, especially the vagus nerve and respiratory activity. By calculating the value, it is possible to know whether the user is in a stress state [29]. To characterize HRV, many authors [30,31] have proposed a variety of hand-crafted HRV metrics that are computed over time intervals between heart rate.

(3) The healing program consisted of breathing, meditation, music, yoga, and stress counseling based on bio-feedback, as shown in Figure 4. Healing breathing, based on bio-feedback, is a cognitive-behavioral therapy strategy based on psychosocial learning theory. When under stress, one’s breathing becomes short and shallow. Practicing strong and deep abdominal breathing when under stress stabilizes one’s emotions. Thus, if the form or rhythm of breathing can be adjusted freely, pulse rate and blood pressure decrease, and the sympathetic nervous system decreases in stimulation (activity), reaching a state of relaxation [27,28]. Second, healing meditation and healing music are used to relax the mood of the user and intended to relieve mental and physical stress by using audiovisual media [29]. Third, healing yoga included a video concerning how to feel comfortable and positive after yoga practice, and it could be accessed at users’ convenience. Lastly, counseling was based on a hidden bulletin board, and it comprised face-to-face, remote counseling. It was conducted by a nurse trained in mental counseling.

In addition, users could check the results of their past examinations. As “other,” a notification and reservation function were added for interactive communication and immediate response using health information and the bulletin board. In addition, the MHapp was divided into six categories: self-management, awareness improvement, technical training, social support, symptom tracking, and data collection [30]. It was developed based on these areas.

### 2.2. Assessment of App Effectiveness

#### 2.2.1. Research Design

We conducted a one-group pretest-posttest study to evaluate the effects of the mobile app that was developed to promote the mental health of adult workers, including their stress, anxiety, depression, emotional labor, well-being, and self-efficacy after 12 weeks of use. We made it possible to use the app at least three times a week. Self-management and information technical training can be select from the app’s healing programs (breathing, music, meditation, yoga), and social support is provided by a professional nurse when applying for mental health counseling. We made it possible to improve awareness, mental health information was provided (once a week), and symptom tracking and data collection were monitored by allowing self-response questionnaires relating to stress, depression, anxiety, etc. PPG measurement results can be self-checked in displayed graphs.

#### 2.2.2. Participants

Participants were selected as adult workers, as normal subjects who did not experience or were not diagnosed treatment for stress, anxiety, and depression, and were selected as high-risk subjects using this app. If participants were on the verge of being treated and were currently receiving treatment, they were excluded.

Participants were adults aged 19 to 64 years who agreed to participate in the research after being informed of its purpose. Participants were recruited by notices and leaflets at companies, health centers, and schools. A total of 108 individuals were recruited; however, 19 were removed because of lack of usage or failure to respond to the survey, and four people taking drugs for depression and anxiety were excluded from the study. Thus, the data from 85 participants were analyzed.

### 2.3. Research Procedure

#### 2.3.1. Ethical Aspects

This research was approved by an Institutional Review Board after a review process concerning research purposes, methodology, participants’ rights and questionnaire composition (No. KHSIRB-19-045).

#### 2.3.2. App Development

The app was developed based on a pilot test in Hwang and Jo [4]. It was conducted for about four months from February to May 2019 based on an analysis of literature, research results, satisfaction, and expert consulting. The program consists of healing music, healing meditation, healing breathing, healing yoga, and counseling. It was designed to enable interactive communication and management information for mental health. After installation, the mobile app was freely used for 12 weeks. Participants were to use it at least twice a week for 10 min. An alarm was set to encourage participants to use the app and induced continued use by providing health education every week.

### 2.4. Data Analysis

The collected data were analyzed using SPSS 23.0. (IBM SPSS Statistics, Armonk, NY, USA) Frequency analysis and technical statistics were conducted for participants’ general characteristics, and the pretest-posttest results were analyzed with paired *t*-tests.

## 3. Results

Participants’ general characteristics are shown in Table 1.

The results concerning app use are shown in Table 2. Concerning the PSS, scores of ≤13, 14–16, 17–18, and ≥19 indicate normal, early stage of stress, high-risk condition, and critical condition, respectively. Prior to the experiment, participants’ mean score was 15.56 ± 3.56 points, indicating early stage of stress. After the experiment, the score increased to 16.80 ± 3.67 points, but remained in the same stage. The ERI results showed no major changes before and after. Depression showed a significant decrease (*p* = 0.043) from 5.65 ± 4.53 before the experiment to 4.80 ± 4.00 after the experiment. There was a significant decrease in anxiety (*p* = 0.003) from 3.92 ± 3.69 to 2.76 ± 2.85 points. Concerning emotional labor, only those under emotional labor were surveyed (*n* = 78). Although there was a decrease before (51.62 ± 22.99 points) and after (55.37 ± 11.82 points), it was non-significant. The stress levels were checked with the PPG sensor immediately after using the app. Before using the app, mean score was 73.46 ± 5.43; after using the app, there was a significant decrease to 64.83 ± 10.07 points (*p* = 0.002).

Satisfaction with the app and awareness of mental health management after using the app are shown in Table 3 and Table 4. Most (81.2%) respondents said they felt the need for mental healthcare after using the app, and 70.8% said they increased their stress-related knowledge by using the app. Attitude to mental health increased (72.9%), request for help (68.2%), and motivation to manage (75.3%) all increased. Concerning satisfaction level after using the app, 40.0% were “satisfied” and 44.7% felt “normal.” Over two-thirds (69.8%) said they would recommend it to others.

## 4. Discussion

This study was designed to develop and evaluate the effectiveness of cognitive behavior-based healing programs using MHapps for mental health management. With the developed app, we measured Korean adults’ stress, depression, anxiety, emotional labor, and well-being. Through this, they could check their mental health status immediately and see the results of their past mental health on a graph. By checking their status in real-time and observing continuous changes, they were to practice breathing, meditation, and yoga through the MHapp [4,31].

The results showed that mobile apps are effective for mental health management. Depression (t = 2.05, *p* = 0.043) and anxiety (t = 3.03, *p* = 0.003) both significantly decreased post-intervention, after the experimental group retained a good or improved condition without depression or anxiety [19,21]. Interventions through mobile apps work immediately without exposing sensitive information [32]. A cognitive-behavioral approach to mood is focused on recognizing and identifying one’s feelings to promote positive emotions and experiences [33]. Many cognitive-behavioral-based programs are effective in adults with high-risk depression [34]. Therefore, it is important for mobile apps developers to be cognizant of people’s emotional state.

Various results were shown concerning participants’ stress levels. Although PSS scores increased (t = 3.43, *p* = 0.001), participants remained in the “early stages of stress” range. The ERI results showed no major changes before and after the intervention. However, for PPG, which shows immediate results after using the app, there was a significant decrease (t = 3.42, *p* = 0.002). Notably, it is difficult to measure stress from a single aspect considering the varied physical changes [35]; therefore, it is necessary to distinguish between perceived chronic stress and acute stress that appears immediately during work or in daily life. However, many studies do not clearly distinguish between acute and chronic stress, and it is difficult to fully assess the individual or common parts between the two [36]. Harkness and Monroe [37] stated that stress measurement is likely to be affected by many causes of bias. They also argued that the measured values should not be considered the same as individuals’ exposure to stress and stress reactions. Given this point, two important characteristics of chronic stress assessments are stability (they represent truly chronic, ongoing conditions) and convergent validity (they are not subjective and correspond to independent measures of similar constructs) [36]. Therefore, app-based interventions are difficult. However, mobile apps appear to be effective for acute or short-lived stress.

Emotional labor and well-being showed little change. Workers’ emotional labor is closely tied to the work environment. Attention is paid to emotional labor because, when workers’ original feelings are managed by the rules of emotion which are artificially constructed to generate profits for the company, the distortion or exaggeration of the nature of workers cause job stress [38]. Emotional labor management can be done at organizational and personal levels; but, in many cases, it must be managed at the organizational level. In 2019, Korea implemented the Special Act on the Protection of Emotional Labor, and the Korea Occupational Safety and Health Agency (KOSHA) prepared guidelines on emotional labor management. It provided that policies on emotional labor management should be prepared, and appropriate service standards and customer response manuals should be developed and distributed, so that regulations on unconditional kindness and response to malicious customers would be applied. It also recommended handling grievances, improving working conditions, and operating health promotion programs. On a personal level, it recommended learning how to control emotions, counseling, communication, positive lifestyles, and various club activities [39]. According to Shim [40], better relationships with others, and higher trust or autonomy, lead to higher levels of well-being. Looking at emotional labor and well-being in this respect, it seems that our results were non-significant, because mobile-based MHapps focus on personal dimensions and cannot affect organizational aspects. However, if the application is used in an organizational manner to expand the operation of health promotion programs and handle grievances, positive results may be expected.

Finally, most participants who used the app recognized the need for mental health management. In addition, most said they would ask for help from experts, which was much higher than the 75.2% of Seoul citizens who said they would take care of it alone or ask for help from acquaintances. The app thus raised awareness of mental health. Further, knowledge and attitude increased, and users were satisfied and would recommend the app. Subjective assessments noted that the app was easy to use and brief. By experiencing the effect of the MHapp, the users affect the continual use of the utility and ease of use of the healthcare app [41,42]. Continued use of mobile apps is fostered by ease of habit, low effort expectancy, and high hedonic motivation MHapps [11,43,44]. Lee and Han [45] showed better results when positive assessments were made about MHapps and when healthcare programs using apps were preferred. Consequently, it is necessary to emphasize the positive aspects of MHapps before using them to users’ willingness to use and to modify and supplement them by providing various content to make it more interesting.

## 5. Conclusions

This study was designed to improve the mental health and well-being of adult Korea workers by creating a mobile app that promotes their health-related quality of life. We confirmed that the app has the potential for mental health improvement. MHapps allow you to freely receive medical services without time and space constraints [46]. Because of this advantage, many MHapps are being released, and consumers are looking for and using apps for mental health [47] to improve their well-being. To efficiently manage the mental health of Korean people, the development and application of effective MHapps is essential. In particular, mental health professionals should serve as leaders in the research, evaluation, and integration of mobile apps [11]. Mental health costs are rising, and self-help intervention, such as those discussed here, are expected to help reduce these costs.

The limitations of this study are its failure to reflect the intervention of chronic stress and emotional labor, and its analysis of the results by applying the app to a single group. When developing or supplementing an app, one needs to think about how much should be included. The app needs to be constantly modified and supplemented to increase its utilization rate. In addition, research is required to verify the effectiveness of the app according to the user characteristics or the results according to the level of stress, anxiety, and depression, and to verify its effect with an experimental group and a control group. Further research is also required on the development of a support system to enhance the effectiveness of a customized mobile app for users concerning their willingness to practice mental health habits.

## Figures and Tables

**Table 1:** General Characteristics of Participants (*N* = 85).

Category			n (%)	Mean ± SD
Gender		Male	22 (25.9)	
		Female	63 (74.1)	
Age (yr)		30 and younger	11 (12.9)	
		31–40	28 (32.9)	
		41–50	36 (42.4)	
		51 and older	6 (11.8)	
Marriage		Unmarried	24 (28.2)	
		Married	61 (71.8)	
Education		High shcool	7 (8.2)	
		College	58 (68.2)	
		Beyond college	20 (23.5)	
Income (millon Won)		<200	8 (9,.4)	
		200 ~ <400	22 (25.9)	
		≥400	55 (64.7)	
BMI		Low weight	1 (1.2)	23.17 ± 2.71
		Normal	51 (60.0)	
		Overweight	9 (10.6))	
		Obesity	24 (28.2)	
Height				164.47 ± 7.06
Weight				62.97 ± 10.04
Working time				8.25 ± 1.85
Blood pressure		DBP		118.53 ± 11.76
		SBP		77.42 ± 9.37
Health Status	Physical health	Not good	10(11.8)	3.3.4 ± 0.76
		Average	41(48.2)	
		Good	34(40.0)	
	Mental health	Not good	8(9.4)	3.46 ± 0.74
		Average	35(41.2)	
		Good	42(49.5)	

DBP = Diastolic blood pressure; SBP = Systolic blood pressure.

**Table 2:** Comparison of Pretest and Posttest *(N* = 85).

Variables		Pre-Test (M ± SD)	Post-Test (M ± SD)	t	*p*
Stress	PSS	15.56 ± 3.56	16.80 ± 3.67	−3.431	0.001
	ERI	35.49 ± 4.55	35.67 ± 4.4.4	−0.423	0.673
Depression		5.65 ± 4.53	4.80 ± 4.00	2.052	0.043
Anxiety		3.92 ± 3.69	2.76 ± 2.85	3.037	0.003
Emotional labor		55.77 ± 11.65	55.37 ± 11.82	0.343	0.732
Well-being		51.62 ± 22.99	49.93 ± 22.92	0.689	0.493
PPG		73.46 ± 5.43	64.83 ± 10.07	3.415	0.002

PSS = Perceived Stress Scale; ERI = effort reward imbalance; PPG = photoplethysmogram.

**Table 3:** Changes in mental health management after using the mental health mobile app (*N* = 78).

Question	Strongly Disagree	Disagree	Agree	Strongly Agree
Recognize the need for mental health management	2(2.4)	7(8.2)	57(67.1)	12(14.1)
Increased mental health management knowledge	2(2.4)	16(18.8)	49(57.9)	11(12.9)
Improving your attitude toward mental health	3(3.5)	13(15.3)	54(63.5)	8(9.4)
Motivation for stress management	1(1.2)	13(15.3)	52(61.2)	12(14.1)
Request for help	2(2.4)	18(21.2)	45(52.9)	13(15.3)
Behavior change	3(3.5)	23(27.1)	46(54.1)	6(7.1)

**Table 4:** Satisfaction of Mental Health Mobile App *(N* = 78).

Question		Item	n (%)
Satisfaction with app		Dissatisfied	6(7.1)
		Usually	38(44.7)
		Satisfied	34(40.0)
Would you recommend the app to others?		Nobody at all	1(1.2)
		Few people	18(21.2)
		To a few people	43(50.6)
		To many people	11(12.9)
		To almost everyone	5(5.9)
Subjective opinion	Benefits	Instant inspection result confirmation and interpretation Healing program that can be applied quickly and in a short time Various programs Easy to use, available anywhere	
	Suggestions	Simplified survey Added alarm function for continuous use and interest Continuous content update Clarity of the user manual	
